# Detection of a common chimeric transcript between human chromosomes 7 and 16

**DOI:** 10.1186/1745-6150-7-49

**Published:** 2012-12-29

**Authors:** Wenwen Fang, Yong Wei, Yibin Kang, Laura F Landweber

**Affiliations:** 1 Department of Molecular Biology, Princeton University, Princeton, NJ, 08544, USA; 2 Department of Ecology and Evolutionary Biology, Princeton University, Princeton, NJ, 08544, USA

**Keywords:** Chimeric transcripts, RNA fusion, *trans*-splicing, Genome rearrangement

## Abstract

**Abstract:**

Interchromosomal chimeric RNA molecules are often transcription products from genomic rearrangement in cancerous cells. Here we report the computational detection of an interchromosomal RNA fusion between *ZC3HAV1L* and *CHMP1A* from RNA-seq data of normal human mammary epithelial cells, and experimental confirmation of the chimeric transcript in multiple human cells and tissues. Our experimental characterization also detected three variants of the *ZC3HAV1L*-*CHMP1A* chimeric RNA, suggesting that these genes are involved in complex splicing. The fusion sequence at the novel exon-exon boundary, and the absence of corresponding DNA rearrangement suggest that this chimeric RNA is likely produced by *trans*-splicing in human cells.

**Reviewers:**

This article was reviewed by Rory Johnson (nominated by Fyodor Kondrashov); Gal Avital and Itai Yanai

## Findings

High-throughput sequencing techniques have allowed characterization of genome and transcriptome catalogs in an unprecedented detail, revealing complex structures of genome rearrangements [1] and transcript networks of chimeric RNAs in human cells [2]. Because genome instability is a hallmark of cancer [3], most studies of genome rearrangements and RNA fusions focus on cancer cells, and some chimeric RNAs appear to result from DNA rearrangements [4-8]. In fact, some studies use RNA-seq data as a guide to annotate genome rearrangement [9]. Several findings, however, suggest that normal human cells also produce chimeric RNA through *trans*-splicing [10-13]. Notably, Li *et al*. demonstrated that in normal endometrial cells, *trans*-splicing produces a chimeric RNA that is identical to a fusion transcript present in endometrial stromal tumor cells [13]. The corresponding chromosomal translocation which may permit production of this chimeric RNA by *cis*-splicing is present in endometrial tumor cells, but not detectable in normal endometrial cells. This hints at the possibility that RNA fusion may even predispose relevant genomic loci to rearrangement [13,14], via RNA-guided DNA recombination, which our lab previously discovered in the ciliate *Oxytricha*[15].

**Figure 1:**
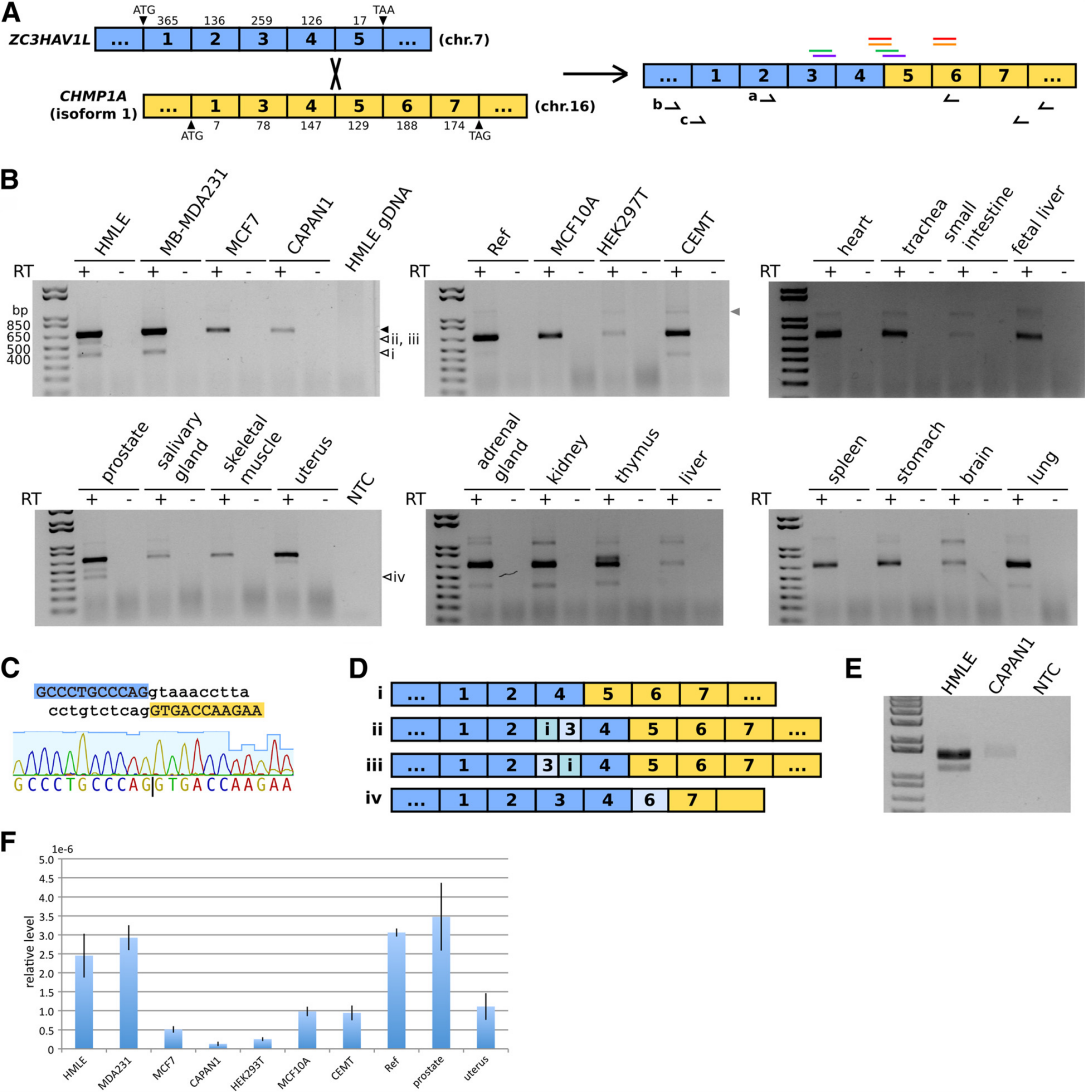
**Detection of *ZC3HAV1L*-*CHMP1A *chimeric RNA in human cells.** (**A**) Schematic representation of the *ZC3HAV1L*-*CHMP1A* chimeric RNA. Blue and yellow boxes indicate exons from *ZC3HAV1L* and *CHMP1A*, respectively. Above the predicted fusion, colored bars indicate paired reads from the ENCODE HMEC RNA-seq data. The same color indicates a read pair supporting the fusion. Arrows below the predicted fusion indicate primer pairs used in PCR analyses (primer pair *a* for PCR in panel B, and primer pairs *b* and *c* for nested PCR in panel E). (**B**) RT-PCR detection of the *ZC3HAV1L*-*CHMP1A* chimeric RNA in human cells and tissues. Filled arrowhead indicates the major predicted fusion; open arrowheads indicate minor alternative chimeric transcripts (i-iv). “RT -” indicates no reverse transcriptase negative controls. “NTC” indicates no template control for PCR analysis. (**C**) Junction sequence of the *ZC3HAV1L*-*CHMP1A* chimeric RNA. Blue and yellow shading highlights sequences from the end of *ZC3HAV1L* exon 4 and start of *CHMP1A* exon 5, respectively. Lower case letters indicate intron sequences. The chromatogram shows Sanger sequencing results at the junction from RT-PCR analysis of HMLE cells. (**D**) Schematic representation of minor alternative *ZC3HAV1L*-*CHMP1A* chimeric RNAs detected from PCR shown in (**B**). Partial exon and introns (“i”) are indicated by different shades of color. Sequence alignment for the major and minor chimeric RNAs is provided in an additional file. (**E**) *ZC3HAV1L*-*CHMP1A* fusion may encode a novel protein. Shown is nested RT-PCR amplification of the predicted fusion coding sequence from HMLE and CAPAN1 cells. The larger band indicates the expected PCR product, whereas the smaller band in HMLE lane indicates the *ZC3HAV1L* exon3^-^ variant. (**F**) *ZC3HAV1L*-*CHMP1A* chimeric RNA levels differ in different human cell and tissue types. The copy number of the RNA fusion between *ZC3HAV1L* exon 4 and *CHMP1A* exon 5 is normalized against *beta-actin* mRNA.

We therefore asked whether we can identify more occurrences of chimeric transcripts, especially those involving genes on separate chromosomes, in normal human cells. We mined high-throughput RNA-seq data from human mammary epithelial cells (HMEC) available from the ENCODE project [16]. The deFuse program [17] predicted one interchromosomal RNA fusion between the genes encoding ZC3HAV1L (zinc finger CCCH-type, antiviral 1-like) and CHMP1A (charged multivesicular body protein 1A), located on chromosomes 7 and 16, respectively (Figure 1A). *ZC3HAV1L* contains 5 exons and encodes a 300 residue protein. *CHMP1A* has two protein-coding transcript isoforms, according to NCBI annotation; transcript variant 1 contains 6 exons encoding a 240 residue protein, and transcript variant 2 contains 7 exons encoding a 196 residue protein, which functions as a tumor suppressor in human kidney and pancreas [18,19]. *CHMP1A* isoform 1 skips exon 2, but has a larger exon 7. Here we used *CHMP1A* isoform 1 as the reference for annotation purposes, because the fusion product we detect contains the larger exon 7 (see below).

Because we initially predicted the presence of the *ZC3HAV1L-CHMP1A* fusion in a breast cell line, we first verified the presence of this chimeric transcript in human mammary cells. Use of a primer pair (Table 1) that amplifies across the predicted *ZC3HAV1L-CHMP1A* fusion junction (from *ZC3HAV1L* exon 2 to *CHMP1A* exon 6) confirms the presence of this fusion at the RNA level in HMLE cells, which are human mammary cells derived from HMEC, but not at the DNA level from matching genomic DNA (Figure 1B). Sequencing of the PCR product verified the fusion junction (Figure 1C). The same PCR analysis suggests that the *ZC3HAV1L-CHMP1A* fusion is present in MCF10A, an immortalized but otherwise normal human mammary epithelial cell line, as well as two human breast cancer cell lines, MB-MDA231 and MCF7. We also detected the fusion in CAPAN1, a pancreatic cancer cell line, human embryonic kidney (HEK) 293 T cells, and CEMT, a human T cell line. In addition, we were able to amplify the fusion RNA from commercially-available human universal reference RNA and a panel of human tissue RNAs (Figure 1B). These results suggest that the *ZC3HAV1L-CHMP1A* chimeric RNA is common across multiple human tissue types, both healthy and diseased.

**Table 1:** Oligonucleotide sequences (5′-3′)

	
ZC3HAV1L_exon2_F	TGGTCTCAATGAAAACCAGCTTCGG
CHMP1A_exon6_R	ATTCTCCTCGGCGATCTGCATGATG
ZC3HAV1L_nested_F	AGCGACCATGGCGGAGCCCA
CHMP1A_nested_R	CACCGCCCAACCTAAAAGAACAGG
ZC3HAV1L_cds_F	ATGGCGGAGCCCACAGTGTGCTCC
CHMP1A_cds_R	CTAAGGCCACGCAGGCCTGGCAG
ZC3HAV1L_qPCR_F	AGAAGCTGGTCCTCTGGCTTCTGT
CHMP1A_qPCR_R	TCACCTGGGCCATATTCTTGGTCA
ACTB_qPCR_F	GCACAGAGCCTCGCCTT
ACTB_qPCR_R	CCTTGCACATGCCGGAG

Curiously, we detected some minor PCR products of different sizes as well. Sequencing revealed that some of them reflect alternative splicing of the *ZC3HAV1L-CHMP1A* chimeric RNA, adding additional complexity to this fusion transcript. A common splicing variant present in multiple tissue types is the full-length chimeric RNA skipping *ZC3HAV1L* exon 3 (Figure 1B, D). Two other minor RT-PCR products suggest splicing between partial intron 2 and exon 3, and partial exon 3 and intron 3 of *ZC3HAV1L*, respectively (Figure 1B, D). Sequence alignments (see Additional file 1) indicate that these alternative splicing events all occur at canonical splicing sites, suggesting that they likely derive from authentic, alternative RNA splicing, rather than an *in vitro* RT-PCR artifact. A fusion product detected from RT-PCR analysis of human prostate RNA fuses *ZC3HAV1L* exon 4 to part of *CHMP1A* exon 6, not at canonical splicing site but between a pair of 5 bp direct repeats at the boundary. Therefore, this may represent either an endogenous activity or just template-switching during the reverse transcription step in our experimental procedure.

The major chimeric, fusion RNA that joins exon 4 of *ZC3HAV1L* to exon 5 of *CHMP1A* preserves the open reading frame. We therefore tested whether the entire open reading frame could be detected from mRNA by nested PCR. The use of primers located upstream and downstream of the predicted start and stop codons in the first round of PCR, and then a nested primer pair between the start and stop codons did amplify a product containing the predicted open reading frame, as well as the *ZC3HAV1L* exon3^-^ version of the transcript, though at much lower levels, consistent with our previous PCR result (Figure 1E). We infer that the major chimeric RNA that joins *ZC3HAV1L* and *CHMP1A* may encode a novel fusion protein. Predicted domains are not available for either fusion partner, however, precluding further structural and functional predictions of this putative fusion protein.

From qPCR analysis, we estimated that the *ZC3HAV1L-CHMP1A* chimeric RNA is present at ~ 0.1 copies per HMLE cell, suggesting that its expression is limited to either a small population of cells, or a transient time window. We also assayed the relative abundance of this chimeric RNA compared to *beta-actin*, a constitutively expressed gene, and found that the relative levels of the *ZC3HAV1L-CHMP1A* chimeric RNA differ in different samples (Figure 1F). This suggests that the production of *ZC3HAV1L-CHMP1A* RNA might be regulated, or it might be a stochastic event.

In summary, we report the discovery of a chimeric RNA between *ZC3HAV1L* and *CHMP1A* in human, located on chromosome 7 and 16, respectively. The fusion occurs at an exon-exon boundary, and was detected both computationally and experimentally from different cells or tissue types. This suggests it is not an artifact from reverse transcription, and is likely an authentic *trans*-splicing product. We also detected three minor variants which also likely result from *trans*-splicing, because the fusion occurs at canonical “GT-AG” splicing sites. The fusion products are present at very low levels, and thus may reflect promiscuous splicing involving *ZC3HAV1L* and *CHMP1A*.

Could such low abundance chimeric RNAs have any function? While the major chimeric RNA that we detected preserves open reading frame and could potentially produce a novel fusion protein or proteins, we propose that such examples of chimeric RNA may also occasionally impact somatic genome rearrangements, facilitating rogue recombination events between the two respective chromosomes [14,15]. The ability of RNA to influence genome remodeling has gained considerable support and interest over the past few years [20], but RNA-guided DNA rearrangement in humans still needs further investigation, emphasizing the importance of detecting more chimeric RNAs and their possible DNA rearrangements in normal or diseased tissue.

### Methods

#### Computational detection of fusion RNAs

HMEC polyA-RNA-seq data from ENCODE project (http://hgdownload.cse.ucsc.edu/goldenPath/hg19/encodeDCC/wgEncodeCshlLongRnaSeq/) were downloaded from UCSC genome browser website. DeFuse 0.4.3 [17] was used to detect fusion transcripts, requiring the presence of two pairs of spanning reads and one split read at the junction. We divided the RNA-seq data into subsets of 10 and 20 million paired-end reads to accommodate computation memory, and the *ZC3HAV1L-CHMP1A* fusion was predicted in two independent subsets of data.

#### RNA and genomic DNA extraction

Total RNA from HMLE, MB-MDA231, MCF7, CAPAN1, MCF10A, HEK293T, and CEMT cells was extracted using RNeasy (Qiagen), and DNase treated with TURBO DNA-free kit (Ambion) following the manufacturer’s instructions. Human reference RNA was purchased from Stratagene, and the human tissue RNA panel was purchased from Clontech. Genomic DNA was extracted using NucleoSpin Tissue (Macherey-Nagel).

#### cDNA synthesis

3.5 μg of RNA was reverse transcribed with SuperScriptIII reverse transcriptase (Invitrogen), in a 20 μl reaction following the oligo(dT) priming protocol.

#### PCR analysis

FastStart High Fidelity PCR system (Roche) was used to amplify fusion product from 1μl of cDNA (equivalent of 175 ng RNA), or 200 ng genomic DNA. To detect the predicted fusion from *ZC3HAV1L* exon 2 to *CHMP1A* exon 6, the following program was used: 95°C 2 min initial denaturing; 95°C 30 s, 58°C 30 s, 72°C 45 s for 36 cycles; 72°C 7 min. To recover the entire coding region by nested PCR, the following program was used. First round: 95°C 2 min initial denaturing; 95°C 30 s, 60°C 30 s, 72°C 90 s for 20 cycles; 72°C 7 min. The PCR reaction was diluted 100 fold, and used in the second round of PCR: 95°C 2 min initial denaturing; 64°C 30 s, 72°C 80 s for 30 cycles; 72°C 7 min.

#### Sanger sequencing of PCR products

PCR products were either sent for direct Sanger sequencing (Genewiz) following Genewiz DNA sequencing instructions, or TOPO-cloned (Invitrogen) for colony PCR and sequencing (Genewiz).

#### qPCR analysis

The 7900HT Fast Real-Time PCR System and SYBR green master mix (Applied Biosystems) were used for qPCR analysis with the default cycling program. Standard curves for each primer pair were generated with five ten-fold serial dilutions of appropriate control plasmids in yeast RNA, allowing absolute quantification of DNA levels. Primer specificity was confirmed by a melt-curve analysis. Each primer pair detected the full range of standards with a correlation of R^2^ > 0.99.

## Reviewers’ comments

### Review by Rory Johnson (Centre de Regulacio Genomica, nominated by Fyodor Kondrashov)

The authors report a new transcript resulting from a fusion of two genes on distinct chromosomes. The range of computational and experimental evidence support the notion that this transcript results from a trans-splicing event rather than from chromosomal translocation or technical artifacts. The manuscript is clear and well presented. I do not have any technical or theoretical arguments with this work. I only have one suggestion: If the fusion transcript has any biological function/phenotype, one might expect that it carries functional domains from its two constituent genes. Therefore, one might expect the fusion junction to occur at linker regions of functional domains, and not interrupting the amino acid sequence in the middle of such a domain. Therefore, I would suggest that the authors comment on where the fusion occurs for both the protein coding sequences with respect to their predicted functional domains, AND include a diagram of the predicted domain structure of the protein encoded by the fusion transcript. E.g. does the fusion carry the zinc finger domain of ZC3HAV1L? What is known about the functions of the two parent protein coding genes?

Author’s response: We did not find any domain annotation for ZC3HAV1L or CHMP1A in Pfam. Despite its name, ZC3HAV1L is actually not a zinc-finger protein. Therefore we did not pursue further the functional predictions of the putative fusion protein. In the revised manuscript we included a comment on this subject.

### Review by Gal Avital and Itai Yanai, Department of Biology, Technion - Israel Institute of Technology

In this report, Fang et al. report a transcript that appears to be trans-spliced between RNAs originating from two different human chromosomes. Four paired-end reads alone appear to support the chimeral transcript in the examined ENCODE RNA-Seq data for one cell type (HMEC). The PCR analysis implicates other cell types, however, do any of these have RNA-Seq datasets in ENCODE to further support this? The article mentions two possible avenues for function following from such a chimera: 1. a protein, and 2. an RNA that might serve to proximally position chromosomes. While the transcript has some of the hallmarks of real transcripts such as the existence of splice variants and an exon-exon boundary, the low expression level does little to support a functional protein. Does the fusion disrupt a functional domain in a putative protein? This can be checked by matching with PFAM. For the second option, structural analysis can be used to query for a stable fold. Overall, while the authors have clearly gone to lengths to detect the chimera in various tissues, a possible function would require additional evidence.

Author’s response: We further analyzed 40 and 20 million paired-end RNA-seq reads for normal lung and placenta from ENCODE data, respectively, but did not find evidence of the ZC3HAV1L-CHMP1A RNA fusion. Our quantitative RT-PCR analysis suggests that levels of the RNA fusion in HMEC are higher than in most other tissues except for prostate, but we have not found appropriate RNA-seq data from normal prostate tissue to use in computational analysis.

We agree that the low level of the chimeric transcript suggests that it more likely has a non-coding function, that could potentially influence genomic rearrangement. We still leave open the possibility of protein-coding function, since the fusion RNA indeed preserves an open reading frame. As we indicated in response to the first review, there is no domain annotation or relevant structural study for these two partner proteins, and therefore we do not further characterize the putative fusion protein at this time.

## Abbreviations

ZC3HAV1L: Zinc finger CCCH-type antiviral 1-like; CHMP1A: Charged multivesicular body protein 1A; HMEC: Human mammary epithelial cell; HEK293T: Human embryonic kidney 293 T; qPCR: Quantitative PCR; gDNA: Genomic DNA.

## Competing interests

The authors declare that they have no competing interests.

## Authors’ contributions

WF performed computational analysis of RNA-seq data, and experimental characterization of the chimeric RNAs. All authors participated in project design and manuscript writing. All authors read and approved the final manuscript.

## Supplementary Material

Additional file 1 **Alignment of the major and minor chimeric transcripts detected in RT-PCR analyses.** The predicted chimeric RNA sequence is indicated in the top row, with annotations of exons below it. Fusion coding sequences (CDS) and the exon3^-^ variant (variant i) are from nested PCR analysis of HMLE cDNA. Variants ii and iii are from PCR analysis of HMLE cDNA, across *ZC3HAV1L* exon 2 and *CHMP1A* exon 6, with partial introns indicated below. Variant iv is from PCR analysis of human prostate cDNA, across *ZC3HAV1L* exon 2 and *CHMP1A* exon 6. The annotations are based on [Genbank: NC_000007 REGION: complement (138710452.138720775) GPC_000000031, NM_080660.3, NM_001083314.2]. Click here for file
