# Supplementary material for: Detection of a common chimeric transcript between human chromosomes 7 and 16
Source: Biol Direct. 2012 Dec 29;7:49. doi: 10.1186/1745-6150-7-49 (PMC3538553; doi:10.1186/1745-6150-7-49)
Supplement: Additional file 1 — Alignment of the major and minor chimeric transcripts detected in RT-PCR analyses. The predicted chimeric RNA sequence is indicated in the top row, with annotations of exons below it. Fusion coding sequences (CDS) and the exon3- variant (variant i) are from nested PCR analysis of HMLE cDNA. Variants ii and iii are from PCR analysis of HMLE cDNA, across ZC3HAV1L exon 2 and CHMP1A exon 6, with partial introns indicated below. Variant iv is from PCR analysis of human prostate cDNA, across ZC3HAV1L exon 2 and CHMP1A exon 6. The annotations are based on [Genbank: NC_000007 REGION: complement (138710452.138720775) GPC_000000031, NM_080660.3, NM_001083314.2]. [file 1745-6150-7-49-S1.pdf]

```
                       1        10        20        30        40        50
1. predicted fusion CDS ATGGCGGAGCCCACAGTGTGCTCCTTCCTCACCAAGGTGCTGTGCGCCCACGGCG
                        ZC3HAV1L-exon1
2. fusion CDS (HMLE)                                                          CG
3. variant i (HMLE)                                                           CG
4. variant ii (HMLE)
5. variant iii (HMLE)
6. variant iv (prostate)

                            60        70        80        90       100       110
1. predicted fusion CDS GCCGCATGTTCCTGAAGGACCTGCGCGGCCACGTGGAGCTGTCGGAGGCCAGGCT
                        ZC3HAV1L-exon1
2. fusion CDS (HMLE)    GCCGCATGTTCCTGAAGGACCTGCGCGGCCACGTGGAGCTGTCGGAGGCCAGGCT
3. variant i (HMLE)     GCCGCATGTTCCTGAAGGACCTGCGCGGCCACGTGGAGCTGTCGGAGGCCAGGCT
4. variant ii (HMLE)
5. variant iii (HMLE)
6. variant iv (prostate)

                                 120       130       140       150       160
1. predicted fusion CDS CCGGGACGTGCTGCAGCGCGCCGGGCCCGAGCGTTTCCTGCTGCAGGAGGTGGAG
                        ZC3HAV1L-exon1
2. fusion CDS (HMLE)    CCGGGACGTGCTGCAGCGCGCCGGGCCCGAGCGTTTCCTGCTGCAGGAGGTGGAG
3. variant i (HMLE)     CCGGGACGTGCTGCAGCGCGCCGGGCCCGAGCGTTTCCTGCTGCAGGAGGTGGAG
4. variant ii (HMLE)
5. variant iii (HMLE)
6. variant iv (prostate)

                            170       180       190       200       210       220
1. predicted fusion CDS ACGCAGGAGGGCCTCGGGGACGCGGAGGCCGAGGCGGCGGCCGGCGCGGTGGGCG
                        ZC3HAV1L-exon1
2. fusion CDS (HMLE)    ACGCAGGAGGGCCTCGGGGACGCGGAGGCCGAGGCGGCGGCCGGCGCGGTGGGCG
3. variant i (HMLE)     ACGCAGGAGGGCCTCGGGGACGCGGAGGCCGAGGCGGCGGCCGGCGCGGTGGGCG
4. variant ii (HMLE)
5. variant iii (HMLE)
6. variant iv (prostate)
```

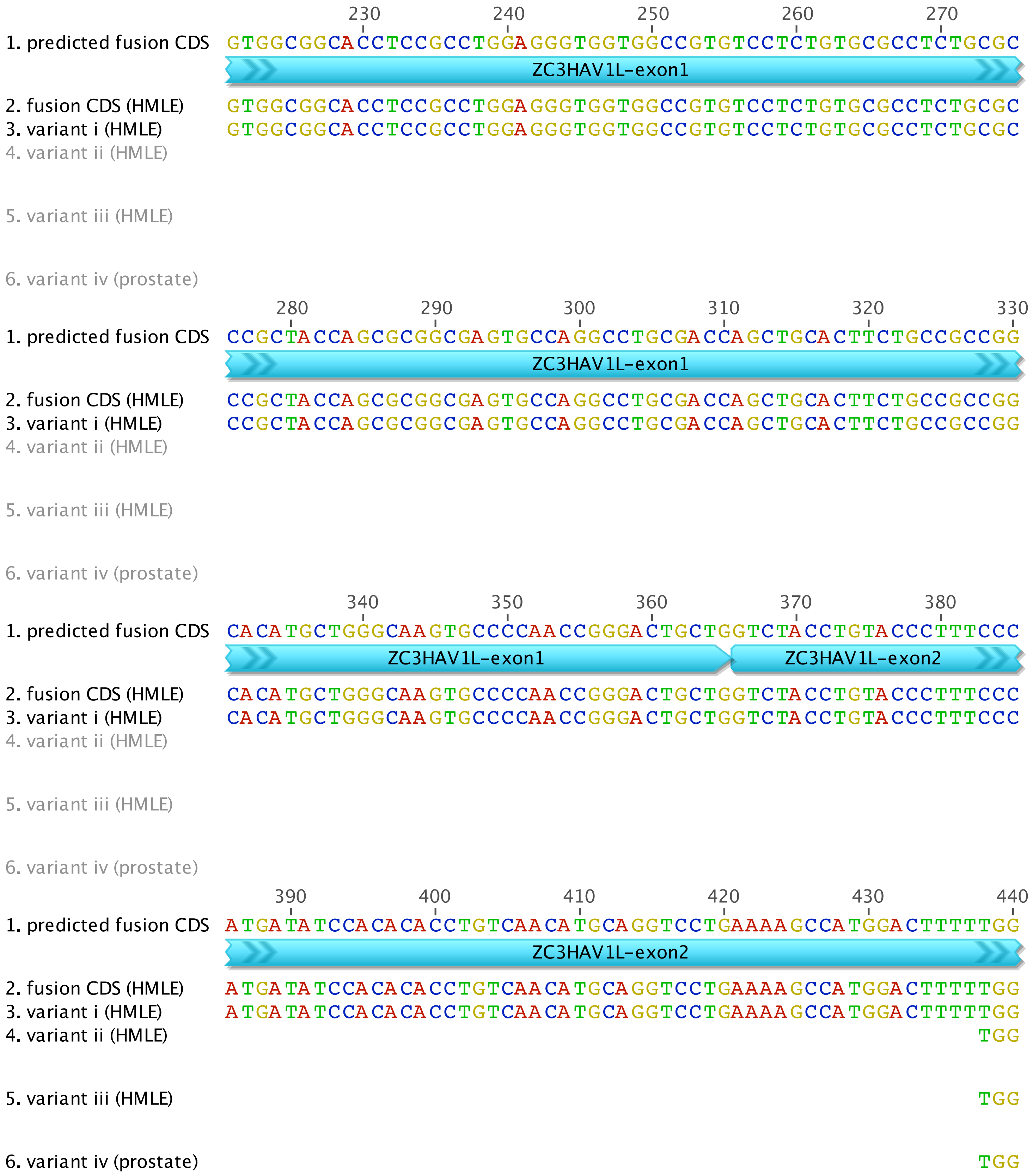
230 240 250 260 270
1. predicted fusion CDS GTGGCGGCACCTCCGCCTGGAGGGTGGTGGCCGTGTCCTCTGTGCGCCTCTGCGC
ZC3HAV1L-exon1
2. fusion CDS (HMLE) GTGGCGGCACCTCCGCCTGGAGGGTGGTGGCCGTGTCCTCTGTGCGCCTCTGCGC
3. variant i (HMLE) GTGGCGGCACCTCCGCCTGGAGGGTGGTGGCCGTGTCCTCTGTGCGCCTCTGCGC
4. variant ii (HMLE)
5. variant iii (HMLE)
6. variant iv (prostate)
280 290 300 310 320 330
1. predicted fusion CDS CCGCTACCAGCGCGGCGAGTGCCAGGCCTGCGACCAGCTGCACTTCTGCCGCCGG
ZC3HAV1L-exon1
2. fusion CDS (HMLE) CCGCTACCAGCGCGGCGAGTGCCAGGCCTGCGACCAGCTGCACTTCTGCCGCCGG
3. variant i (HMLE) CCGCTACCAGCGCGGCGAGTGCCAGGCCTGCGACCAGCTGCACTTCTGCCGCCGG
4. variant ii (HMLE)
5. variant iii (HMLE)
6. variant iv (prostate)
340 350 360 370 380
1. predicted fusion CDS CACATGCTGGGCAAGTGCCCCAACCGGGACTGCTGGTCTACCTGTACCCTTTCCC
ZC3HAV1L-exon1
ZC3HAV1L-exon2
2. fusion CDS (HMLE) CACATGCTGGGCAAGTGCCCCAACCGGGACTGCTGGTCTACCTGTACCCTTTCCC
3. variant i (HMLE) CACATGCTGGGCAAGTGCCCCAACCGGGACTGCTGGTCTACCTGTACCCTTTCCC
4. variant ii (HMLE)
5. variant iii (HMLE)
6. variant iv (prostate)
390 400 410 420 430 440
1. predicted fusion CDS ATGATATCCACACACCTGTCAACATGCAGGTCCTGAAAAGCCATGGACTTTTTGG
ZC3HAV1L-exon2
2. fusion CDS (HMLE) ATGATATCCACACACCTGTCAACATGCAGGTCCTGAAAAGCCATGGACTTTTTGG
3. variant i (HMLE) ATGATATCCACACACCTGTCAACATGCAGGTCCTGAAAAGCCATGGACTTTTTGG
4. variant ii (HMLE) TGG
5. variant iii (HMLE) TGG
6. variant iv (prostate) TGG

```
                                    450       460       470       480       490
1. predicted fusion CDS     TCTCAATGAAAACCAGCTTCGGATCCTGCTTTTGCAGAATGACCCCTGTCTTTTA
                            [ZC3HAV1L-exon2]
2. fusion CDS (HMLE)        TCTCAATGAAAACCAGCTTCGGATCCTGCTTTTGCAGAATGACCCCTGTCTTTTA
3. variant i (HMLE)         TCTCAATGAAAACCAGCTTCGGATCCTGCTTTTGCAGAATGACCCCTGTCTTTTA
4. variant ii (HMLE)        TCTCAATGAAAACCAGCTTCGGATCCTGCTTTTGCAGAATGACCCCTGTCTTTTA

5. variant iii (HMLE)       TCTCAATGAAAACCAGCTTCGGATCCTGCTTTTGCAGAATGACCCCTGTCTTTTA

6. variant iv (prostate)    TCTCAATGAAAACCAGCTTCGGATCCTGCTTTTGCAGAATGACCCCTGTCTTTTA

                                500       510       520       530       540       550
1. predicted fusion CDS     CCAGAGGTCTGTTTGCTCTACAACAAAGGGGAAGCCCTGTATGGCTACTGCAACC
                                  [ZC3HAV1L exon3]
2. fusion CDS (HMLE)        CCAGAGGTCTGTTTGCTCTACAACAAAGGGGAAGCCCTGTATGGCTACTGCAACC
3. variant i (HMLE)         CCAGAG-------------------------------------------------
4. variant ii (HMLE)        CCAGAGCCTAAAAATGCCCTCGTCTGGATCATAAATTAGATCTTTGTGCCACCCG
                                  [partial intron 2]
5. variant iii (HMLE)       CCAGAG-------------------------------------------------

6. variant iv (prostate)    CCAGAGGTCTGTTTGCTCTACAACAAAGGGGAAGCCCTGTATGGCTACTGCAACC

                                    560       570       580       590       600
1. predicted fusion CDS     TCAAGGATAAATGCAACAAGTTTCATGTGTGCAAATCCTTTGTGAAAGGAGAATG
                            [ZC3HAV1L exon3]
2. fusion CDS (HMLE)        TCAAGGATAAATGCAACAAGTTTCATGTGTGCAAATCCTTTGTGAAAGGAGAATG
3. variant i (HMLE)         -------------------------------------------------------
4. variant ii (HMLE)        TTGAAACTGGTTTCCCTGGAAGCAGAGCCTGAGATAGAGACC-------------
                            [partial intron 2]
5. variant iii (HMLE)       -------------------------------------------------------

6. variant iv (prostate)    TCAAGGATAAATGCAACAAGTTTCATGTGTGCAAATCCTTTGTGAAAGGAGAATG

                                610       620       630       640       650       660
1. predicted fusion CDS     CAAACTTCAGACCTGCAAACGGTCCCATCAGCTTATCCATGCTGCATCTTTGAAG
                            [ZC3HAV1L exon3]
2. fusion CDS (HMLE)        CAAACTTCAGACCTGCAAACGGTCCCATCAGCTTATCCATGCTGCATCTTTGAAG
3. variant i (HMLE)         -------------------------------------------------------
4. variant ii (HMLE)        -------------------------------------------------------

5. variant iii (HMLE)       -------------------------------------------------------

6. variant iv (prostate)    CAAACTTCAGACCTGCAAACGGTCCCATCAGCTTATCCATGCTGCATCTTTGAAG
```

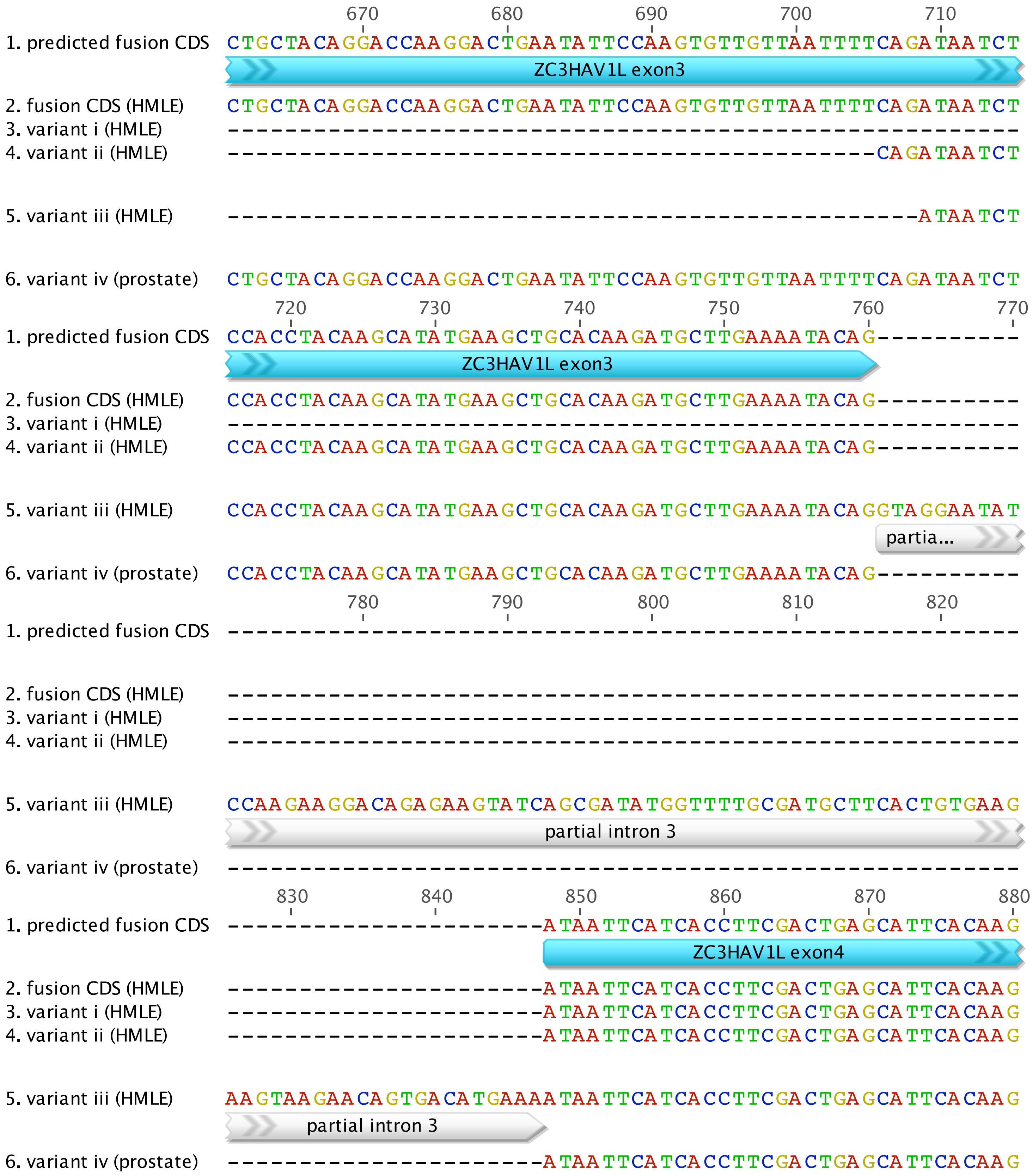
670 680 690 700 710
1. predicted fusion CDS CTGCTACAGGACCAAGGACTGAATATTCCAAGTGTTGTTAATTTTCAGATAATCT
ZC3HAV1L exon3
2. fusion CDS (HMLE) CTGCTACAGGACCAAGGACTGAATATTCCAAGTGTTGTTAATTTTCAGATAATCT
3. variant i (HMLE) --
4. variant ii (HMLE) ---CAGATAATCT
5. variant iii (HMLE) ---ATAATCT
6. variant iv (prostate) CTGCTACAGGACCAAGGACTGAATATTCCAAGTGTTGTTAATTTTCAGATAATCT
720 730 740 750 760 770
1. predicted fusion CDS CCACCTACAAGCATATGAAGCTGCACAAGATGCTTGAAAATACAG----------
ZC3HAV1L exon3
2. fusion CDS (HMLE) CCACCTACAAGCATATGAAGCTGCACAAGATGCTTGAAAATACAG----------
3. variant i (HMLE) --
4. variant ii (HMLE) CCACCTACAAGCATATGAAGCTGCACAAGATGCTTGAAAATACAG----------
5. variant iii (HMLE) CCACCTACAAGCATATGAAGCTGCACAAGATGCTTGAAAATACAGGTAGGAATAT
partia...
6. variant iv (prostate) CCACCTACAAGCATATGAAGCTGCACAAGATGCTTGAAAATACAG----------
780 790 800 810 820
1. predicted fusion CDS --
2. fusion CDS (HMLE) --
3. variant i (HMLE) --
4. variant ii (HMLE) --
5. variant iii (HMLE) CCAAGAAGGACAGAGAAGTATCAGCGATATGGTTTTGCGATGCTTCACTGTGAAG
partial intron 3
6. variant iv (prostate) --
830 840 850 860 870 880
1. predicted fusion CDS -----------------------ATAATTCATCACCTTCGACTGAGCATTCACAAG
ZC3HAV1L exon4
2. fusion CDS (HMLE) -----------------------ATAATTCATCACCTTCGACTGAGCATTCACAAG
3. variant i (HMLE) -----------------------ATAATTCATCACCTTCGACTGAGCATTCACAAG
4. variant ii (HMLE) -----------------------ATAATTCATCACCTTCGACTGAGCATTCACAAG
5. variant iii (HMLE) AAGTAAGAACAGTGACATGAAAAATAATTCATCACCTTCGACTGAGCATTCACAAG
partial intron 3
6. variant iv (prostate) -----------------------ATAATTCATCACCTTCGACTGAGCATTCACAAG

```
                            890       900       910       920       930
1. predicted fusion CDS  GCCTTGAGAAGCAAGGAGTGCACGCAGCTGGAGCTGCAGAAGCTGGTCCTCTGGC
                         [ZC3HAV1L exon4]
2. fusion CDS (HMLE)     GCCTTGAGAAGCAAGGAGTGCACGCAGCTGGAGCTGCAGAAGCTGGTCCTCTGGC
3. variant i (HMLE)      GCCTTGAGAAGCAAGGAGTGCACGCAGCTGGAGCTGCAGAAGCTGGTCCTCTGGC
4. variant ii (HMLE)     GCCTTGAGAAGCAAGGAGTGCACGCAGCTGGAGCTGCAGAAGCTGGTCCTCTGGC

5. variant iii (HMLE)    GCCTTGAGAAGCAAGGAGTGCACGCAGCTGGAGCTGCAGAAGCTGGTCCTCTGGC

6. variant iv (prostate) GCCTTGAGAAGCAAGGAGTGCACGCAGCTGGAGCTGCAGAAGCTGGTCCTCTGGC

                         940       950       960       970       980       990
1. predicted fusion CDS  TTCTGTCCCTGCTCAGTCGGCCAAGAAGCCCTGCCCAGGTGACCAAGAATATGGC
                         [ZC3HAV1L exon4]                    [CHMP1A exon5]
2. fusion CDS (HMLE)     TTCTGTCCCTGCTCAGTCGGCCAAGAAGCCCTGCCCAGGTGACCAAGAATATGGC
3. variant i (HMLE)      TTCTGTCCCTGCTCAGTCGGCCAAGAAGCCCTGCCCAGGTGACCAAGAATATGGC
4. variant ii (HMLE)     TTCTGTCCCTGCTCAGTCGGCCAAGAAGCCCTGCCCAGGTGACCAAGAATATGGC

5. variant iii (HMLE)    TTCTGTCCCTGCTCAGTCGGCCAAGAAGCCCTGCCCAGGTGACCAAGAATATGGC

6. variant iv (prostate) TTCTGTCCCTGCTCAGTCGGCCAAGAAGCCCTG----------------------

                         1,000     1,010     1,020     1,030     1,040
1. predicted fusion CDS  CCAGGTGACCAAAGCCCTGGACAAGGCCCTGAGCACCATGGACCTGCAGAAGGTC
                         [CHMP1A exon5]
2. fusion CDS (HMLE)     CCAGGTGACCAAAGCCCTGGACAAGGCCCTGAGCACCATGGACCTGCAGAAGGTC
3. variant i (HMLE)      CCAGGTGACCAAAGCCCTGGACAAGGCCCTGAGCACCATGGACCTGCAGAAGGTC
4. variant ii (HMLE)     CCAGGTGACCAAAGCCCTGGACAAGGCCCTGAGCACCATGGACCTGCAGAAGGTC

5. variant iii (HMLE)    CTAGGTGACCAAAGCCCTGGACAAGGCCCTGAGCACCATGGACCTGCAGAAGGTC

6. variant iv (prostate) -------------------------------------------------------

                         1,050     1,060     1,070     1,080     1,090     1,100
1. predicted fusion CDS  TCCTCAGTGATGGACAGGTTCGAGCAGCAGGTGCAGAACCTGGACGTCCATACAT
                         [CHMP1A exon5]
2. fusion CDS (HMLE)     TCCTCAGTGATGGACAGGTTCGAGCAGCAGGTGCAGAACCTGGACGTCCATACAT
3. variant i (HMLE)      TCCTCAGTGATGGACAGGTTCGAGCAGCAGGTGCAGAACCTGGACGTCCATACAT
4. variant ii (HMLE)     TCCTCAGTGATGGACAGGTTCGAGCAGCAGGTGCAGAACCTGGACGTCCATACAT

5. variant iii (HMLE)    TCCTCAGTGATGGACAGGTTCGAGCAGCAGGTGCAGAACCTGGGCGTCCATACAT

6. variant iv (prostate) -------------------------------------------------------
```

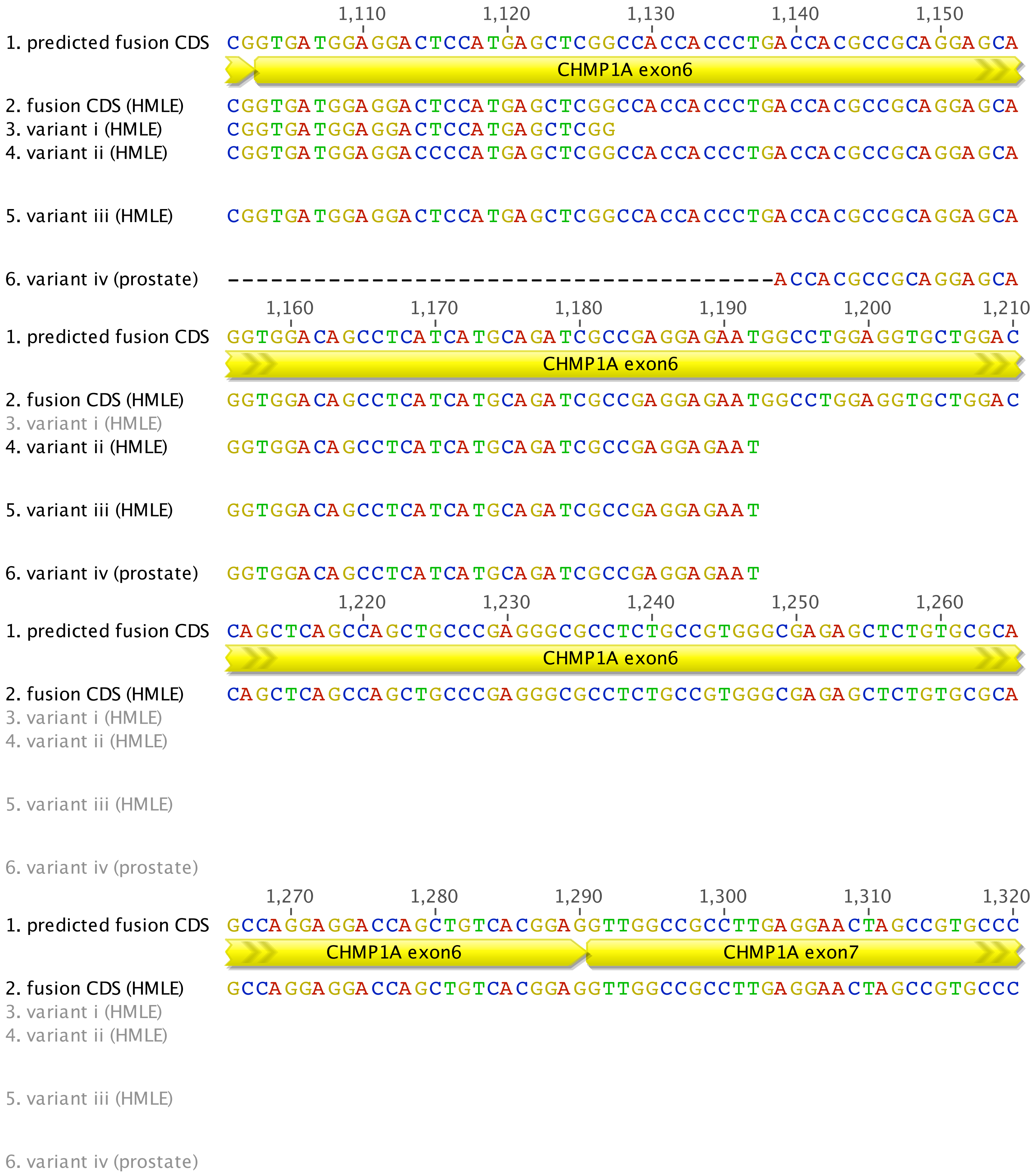
1,110 1,120 1,130 1,140 1,150
1. predicted fusion CDS CGGTGATGGAGGACTCCATGAGCTCGGCCACCACCCTGACCACGCCGCAGGAGCA
CHMP1A exon6
2. fusion CDS (HMLE) CGGTGATGGAGGACTCCATGAGCTCGGCCACCACCCTGACCACGCCGCAGGAGCA
3. variant i (HMLE) CGGTGATGGAGGACTCCATGAGCTCGG
4. variant ii (HMLE) CGGTGATGGAGGACCCCATGAGCTCGGCCACCACCCTGACCACGCCGCAGGAGCA
5. variant iii (HMLE) CGGTGATGGAGGACTCCATGAGCTCGGCCACCACCCTGACCACGCCGCAGGAGCA
6. variant iv (prostate) --------------------------------------ACCACGCCGCAGGAGCA
1,160 1,170 1,180 1,190 1,200 1,210
1. predicted fusion CDS GGTGGACAGCCTCATCATGCAGATCGCCGAGGAGAATGGCCTGGAGGTGCTGGAC
CHMP1A exon6
2. fusion CDS (HMLE) GGTGGACAGCCTCATCATGCAGATCGCCGAGGAGAATGGCCTGGAGGTGCTGGAC
3. variant i (HMLE)
4. variant ii (HMLE) GGTGGACAGCCTCATCATGCAGATCGCCGAGGAGAAT
5. variant iii (HMLE) GGTGGACAGCCTCATCATGCAGATCGCCGAGGAGAAT
6. variant iv (prostate) GGTGGACAGCCTCATCATGCAGATCGCCGAGGAGAAT
1,220 1,230 1,240 1,250 1,260
1. predicted fusion CDS CAGCTCAGCCAGCTGCCCGAGGGCGCCTCTGCCGTGGGCGAGAGCTCTGTGCGCA
CHMP1A exon6
2. fusion CDS (HMLE) CAGCTCAGCCAGCTGCCCGAGGGCGCCTCTGCCGTGGGCGAGAGCTCTGTGCGCA
3. variant i (HMLE)
4. variant ii (HMLE)
5. variant iii (HMLE)
6. variant iv (prostate)
1,270 1,280 1,290 1,300 1,310 1,320
1. predicted fusion CDS GCCAGGAGGACCAGCTGTCACGGAGGTTGGCCGCCTTGAGGAACTAGCCGTGCCC
CHMP1A exon6
CHMP1A exon7
2. fusion CDS (HMLE) GCCAGGAGGACCAGCTGTCACGGAGGTTGGCCGCCTTGAGGAACTAGCCGTGCCC
3. variant i (HMLE)
4. variant ii (HMLE)
5. variant iii (HMLE)
6. variant iv (prostate)

1,330 1,340 1,350 1,360 1,370

1. predicted fusion CDS CGCCGGTGTGCACCGCCTCTGCCCCGTGATGTGCTGGAAGGCTCCTGTCCTCTCC

CHMP1A exon7

2. fusion CDS (HMLE) CGCCGGTGTGCACCGCCTCTGCCCCGTGATGTGCTGGAAGGCTCCTGTCCTCTCC

3. variant i (HMLE)

4. variant ii (HMLE)

5. variant iii (HMLE)

6. variant iv (prostate)

1,380 1,390 1,400 1,410 1,420 1,430

1. predicted fusion CDS CCACCGCGTCTTGCCTTTGTGCTGACCCCGCGGGGCTGCGGCCGGCAGCCACTCT

CHMP1A exon7

2. fusion CDS (HMLE) CCACCGCGTCTTGCCTTTGTGCTGACCCCGCGGGGCTGCGGCCG

3. variant i (HMLE)

4. variant ii (HMLE)

5. variant iii (HMLE)

6. variant iv (prostate)

1,440 1,450 1,460 1,464

1. predicted fusion CDS GCGTCTCTCACCTGCCAGGCCTGCGTGGCCTTAG

CHMP1A exon7

2. fusion CDS (HMLE)

3. variant i (HMLE)

4. variant ii (HMLE)

5. variant iii (HMLE)

6. variant iv (prostate)
